# Phosphorylated fraction of H2AX as a measurement for DNA damage in cancer cells and potential applications of a novel assay

**DOI:** 10.1371/journal.pone.0171582

**Published:** 2017-02-03

**Authors:** Jiuping Ji, Yiping Zhang, Christophe E. Redon, William C. Reinhold, Alice P. Chen, Laura K. Fogli, Susan L. Holbeck, Ralph E. Parchment, Melinda Hollingshead, Joseph E. Tomaszewski, Quentin Dudon, Yves Pommier, James H. Doroshow, William M. Bonner

**Affiliations:** 1 Applied/Developmental Research Directorate, Leidos Biomedical Research, Inc., Frederick National Laboratory for Cancer Research, Frederick, Maryland, United States of America; 2 Developmental Therapeutics Branch, Center for Cancer Research, National Cancer Institute, Bethesda, Maryland, United States of America; 3 Early Clinical Trials Development Program, Division of Cancer Treatment and Diagnosis, National Cancer Institute, Bethesda, Maryland, United States of America; 4 Division of Cancer Treatment and Diagnosis, National Cancer Institute, Bethesda, Maryland, United States of America; The University of Hong Kong, HONG KONG

## Abstract

Phosphorylated H2AX (γ-H2AX) is a sensitive marker for DNA double-strand breaks (DSBs), but the variability of H2AX expression in different cell and tissue types makes it difficult to interpret the meaning of the γ-H2AX level. Furthermore, the assays commonly used for γ-H2AX detection utilize laborious and low-throughput microscopy-based methods. We describe here an ELISA assay that measures both phosphorylated H2AX and total H2AX absolute amounts to determine the percentage of γ-H2AX, providing a normalized value representative of the amount of DNA damage. We demonstrate the utility of the assay to measure DSBs introduced by either ionizing radiation or DNA-damaging agents in cultured cells and in xenograft models. Furthermore, utilizing the NCI-60 cancer cell line panel, we show a correlation between the basal fraction of γ-H2AX and cellular mutation levels. This additional application highlights the ability of the assay to measure γ-H2AX levels in many extracts at once, making it possible to correlate findings with other cellular characteristics. Overall, the γ-H2AX ELISA represents a novel approach to quantifying DNA damage, which may lead to a better understanding of mutagenic pathways in cancer and provide a useful biomarker for monitoring the effectiveness of DNA-damaging anticancer agents.

## Introduction

The accurate measurement of DNA double-strand breaks (DSBs) has become crucial in both basic research and clinical studies. Assessment of DNA damage is relevant to various areas of research, including aging, DNA repair pathways, and apoptosis [1]. Understanding the extent of DNA breakage is especially relevant to the study of tumorigenesis, as many cancers are known to have mutations in DNA damage response pathways that act to repair DSBs, and these defects contribute to the genomic instability that drives tumor development [2]. Furthermore, many anticancer agents kill tumor cells by introducing DSBs and activating cell death pathways, making the measurement of DSBs useful in evaluating tumor response to treatment [3–5].

One of the earliest events in the response to nascent DNA damage in humans is the phosphorylation of histone H2A variant H2AX on a serine four residues from the C-terminus (residue 139) to form γ-H2AX [6]. The response is highly amplified, with the phosphorylation of many H2AX molecules flanking the DSB site over a period of 10 to 30 minutes after DNA damage induction [7]. In the last decade, γ-H2AX has become a powerful biomarker for the quantification of DSB levels in cells and tissues [3], [8–11].

The detection of γ-H2AX relies on immunological techniques using specific antibodies, either in intact cells and tissues or in cell and tissue lysates. In intact fixed cells, the phosphorylated H2AX molecules appear as a focus at the break site in the nucleus, with the number of foci per nucleus being proportional to the amount of induced DNA damage. While microscopy-based foci quantitation is the most sensitive assay to measure DSB levels, it is also the most labor-intensive and the least suitable for high-throughput applications. Tissue samples must be individually prepared for immunofluorescence microscopy, and images of hundreds or thousands of cells must be processed to enumerate γ-H2AX foci or measure γ-H2AX signal intensity [12]. The other option is flow cytometry, which does allow for rapid quantification of γ-H2AX levels in cell samples but is low-throughput and limited in sensitivity [13].

Measuring γ-H2AX levels in lysates can be performed through Western blotting or the enzyme-linked immunosorbent assay (ELISA). Western blotting is unable to detect subtle differences in γ-H2AX levels, making this technique impractical for most clinical samples [14]. In contrast, the higher sensitivity of the ELISA to monitor γ-H2AX kinetics has led to its use in a recent clinical trial, demonstrating the utility of the ELISA technique and the value in optimizing the γ-H2AX assay [8], [15]. The ELISA method also provides an opportunity to evaluate total H2AX levels in cell and tissue lysates. Cellular H2AX content is cell- or tissue-specific and can vary over a large spectrum, ranging from 2 to 25% of total H2A [6]. While histones are essential for the condensation and protection of DNA in all eukaryotic cells, changes in histone composition occur during normal cell differentiation [16], [17], cellular reprogramming [18], and cancer progression [19–21], and alteration in histone expression has been observed in several different types of cancer [20], [22]. The plots generated based on data obtained from the NCI-60 cancer cell line panel [23] and the Cancer Cell Line Encyclopedia (CCLE) [24] show that while the number of copies of the H2AX gene roughly correlates with the number of mRNA transcripts (correlation coefficients of 0.75 for NCI-60 and 0.45 for CCLE), the association is not strong enough to accurately predict H2AX protein expression based on gene copy number in tumor cells (S1A and S1B Fig).

The complex regulation of H2AX levels can complicate the study of γ-H2AX and the cellular response to DNA damage. Due to the variability in H2AX expression, meaningful comparisons of γ-H2AX levels among different cell and tissue types can only be made after measurements are normalized to total H2AX levels. This paper describes the first sensitive and improved ELISA for the quantification of phosphorylated H2AX relative to the amount of total H2AX. We demonstrate here the ability of this assay to accurately measure DSBs induced by ionizing radiation or DNA-damaging agents in both cells and tissues. In addition, total H2AX acts as an internal control for losses during sample preparation, alleviating the need for other normalization measurements such as protein concentration or cell counts. Furthermore, utilizing the NCI-60 human tumor cell lines, we show that there is a correlation between the baseline fraction of phosphorylated H2AX and mutational load in the various cell lines. Our results highlight the potential of the γ-H2AX ELISA to improve the study of mutagenesis and DNA damage response pathways in cancer.

## Materials and methods

### Ethics statement

The Frederick National Laboratory for Cancer Research is accredited by Association for Assessment and Accreditation of Laboratory Animal Care International and follows the USPHS Policy for the Care and Use of Laboratory Animals. All the studies were conducted according to an approved animal care and use committee protocol in accordance with the procedures outlined in the ‘‘Guide for Care and Use of Laboratory Animals” (National Research Council; 1996; National Academy Press; Washington, DC).

### Antibodies and lab reagents

Phospho-H2AX (clone JBW301; specific to phosphorylation on S139) mouse monoclonal antibody was obtained from Millipore (Billerica, MA; cat # 05–636) and H2A monoclonal antibody (clone 4F10) was obtained from Novus (Littleton, CO; cat # H00008334-M01). Antibody reactivity and specificity were verified through Western blots performed in-house (data not shown). H2AX rabbit polyclonal antibody was obtained from Abcam (Cambridge, MA; cat # ab10475). The HRP-conjugated anti-rabbit antibody used for recognition of the H2AX rabbit antibody was from KPL (Gaithersburg, MD; cat # 074-15-061), and the SuperSignal ELISA Pico Chemiluminescent substrate used was from Pierce Thermo Scientific (Rockford, IL; cat # 37070). The following buffers and serums were used: carbonate-bicarbonate coating buffer, pH 9.6 from Sigma (St. Louis, MO; cat # C3041-50CAP); SuperBlock in PBS from Pierce Thermo Scientific (cat # 37535); cell extraction buffer from Invitrogen (Carlsbad, CA; cat # FNN0011); bovine serum albumin (BSA) from Sigma (cat # A7030); and mouse serum from Sigma (cat # M5905). 10X Phosphate Buffered Saline was obtained from Invitrogen (cat # 70013–073) and 20% SDS from Roche (Indianapolis, IN; cat # 11 666 924 001). Protease Inhibitor Cocktail and Phosphatase Inhibitor Cocktail tablets (PhosStop) were from Roche (cat #s 11 697 498 001, 04 906 837 001). Phenylmethanesulfonyl fluoride (PMSF) was from Sigma (cat # 93482-50ML-F). γ-H2AX standard was synthesized by Invitrogen, full length total H2AX recombinant standard was from Axxora (San Diego, CA; cat # ALX-201-176-M005), and full length H2A recombinant protein was from EMD Millipore (cat # 14–493).

### Cell lines and treatment in vitro

Frozen cell pellets and active cultures from the NCI-60 cell bank were provided by the NCI Division of Cancer Treatment and Diagnosis (Bethesda, MD). Additional cell lines were acquired from ATCC (Manassas, VA) and cultured at the National Clinical Target Validation Laboratory (NCTVL) following the recommended cell culture procedures (RPMI 1640 + 10% fetal bovine serum + 1% L-Glutamine; 37°C, 5% CO_2_, >70% humidity). Cells were harvested when an 85% to 95% monolayer was reached, approximately 3 to 4 days after plating.

Cells were exposed to either radiation or the indicated agents while in the exponential growth phase. The Mark-1 irradiator (JL Shepherd & Associates, San Fernando, CA) containing a 137Cs source was used for cell irradiation. Cells were irradiated at room temperature and then incubated for 30 minutes at 37°C under standard conditions before protein extraction. SN38 (NSC 673596), LMP744 (NSC 706744), cisplatin (NSC 119875), etoposide (NSC 141540), olaparib (NSC 747856), vorinostat (NSC 701852), erlotinib (NSC 718781), and romidepsin (NSC 630176) were from the Developmental Therapeutics Program (DTP) Drug Repository in the Division of Cancer Treatment and Diagnosis at NCI. Specific drug concentrations for *in vitro* studies are specified in the figure legends for each experiment.

### Cell extract preparation

NCI-60 cells and liquid biopsies were lysed in cell extraction buffer (Invitrogen) with protease and phosphatase inhibitor cocktails (Roche) and 1% PMSF (Sigma) on ice for 30 minutes. SDS was then added to a final concentration of 1%. Cell lysates were heated for 5 minutes at 100°C and then centrifuged at 16,000 x g for 5 minutes.

### Animal models

Mice were housed in sterile, filter-capped polycarbonate cages (Allentown Caging, Allentown, NJ), maintained in a barrier facility on a 12-hour light/dark cycle, and were provided sterilized food and water ad libitum. Female athymic nude mice (Frederick National Laboratory for Cancer Research Animal Production Program) were implanted with the human melanoma cell line A375 as previously reported [25]. All cell lines were purchased from ATCC. Tumors were maintained by serial *in vivo* passage using tumor fragment transplantation when the donor tumors reached 10 to 15 mm in diameter.

Animals were staged to the desired tumor weight (200 mg; between 7 and 8 mm in diameter), calculated using the following formula: weight (mg) = (tumor length x [tumor width]^2^)/2. Each animal received a single drug dose and then was sacrificed within 24 hours of dosing for sample collection. The tumors were not repeatedly measured as the animals were not studied longitudinally.

Mice were sacrificed by IACUC approved methods (consistent with AVMA Guidelines for Euthanasia), including induction and maintenance of isoflurane anesthesia with exsanguination or exposure to carbon dioxide for a minimum of 10 minutes followed by additional observation or cervical dislocation. All animals were monitored daily for clinical signs of distress (e.g., hunched posture, roughened haircoat, inactivity, general body condition, weight loss, inappetance, self-isolation). Animals showing signs of distress were treated with wet feed/hydrogel if signs were mild (weight loss less than 20%; only 1 clinical sign apparent) or humanely euthanized if signs were more than mild.

### Drug administration in mice

Olaparib (NSC 747856) and CPT11 (irinotecan; NSC 616348) were provided by the Developmental Therapeutics Program (DTP) Drug Repository in the Division of Cancer Treatment and Diagnosis at NCI. Xenograft mice were treated with olaparib at 50 mg/kg through intraperitoneal injection and with CPT11 at 7.5 mg/kg through intravenous injection. Treatment protocols were the same as previously described [12]. Mice were randomized before initiation of treatment using a commercial software program (Study Director, Studylog Systems, Inc.).

### Xenograft collection

Cohorts of at least three mice per treatment group were anesthetized with isoflurane 6 hours post-treatment, and xenograft tumor pieces were collected by resection once surgical anesthesia was reached (no toe pinch response). Specimens were flash-frozen, dry, in pre-cooled o-ring screw-cap conical vials as previously described [25]. Sampling was timed from the beginning of drug administration. Frozen tumor biopsies were stored in liquid nitrogen (preferred) or -80°C until processing.

### Tumor extract preparation

Tumor biopsies (10–50 mg) were homogenized on ice using a Bio-Gen Pro200 homogenizer (ProScientific, Oxford, CT) in 300–500 μL of cell extraction buffer containing protease inhibitor and 1% PMSF. The extract was incubated on ice for 20 minutes, and SDS was then added to the extract at room temperature to reach a final concentration of 1%. The extract was heated to 100°C for 5 minutes and then centrifuged at 16,000 x g at 4°C for 10 minutes. The supernatant was collected as the tumor lysate. Protein concentration was determined with the Pierce BCA kit (Thermo Scientific).

### γ-H2AX and H2AX sandwich ELISA assay

Pierce Reacti-bind 96-well plates (Thermo Scientific) were coated with phospho-H2AX monoclonal antibody for the γ-H2AX immunoassay and H2A monoclonal antibody for the total H2AX immunoassay. The coated plate was blocked with 2% BSA in PBS for 1 hour at 37°C. 50 μL 2% BSA in PBS was loaded prior to loading 25 μL of each sample. For the total H2AX ELISA, 100 ng/well protamine was also added. The plate was incubated overnight at 4°C. After incubation, H2AX rabbit polyclonal antibody against the H2A core was used for detection, and then HRP-conjugated affinity-purified goat anti-rabbit antibody was added. In the presence of freshly prepared SuperSignal ELISA Pico Chemiluminescent Substrate, signal from 96-well plate was immediately read on an Infinite M200 plate reader (Tecan US, Research Triangle Park, NC). The relative light unit (RLU) values were used to generate the γ-H2AX or total H2AX standard curve. The percentage of γ-H2AX to total H2AX level for each cell extract was then determined. Lab working protocols with detailed instructions can be found on the Division of Cancer Treatment and Diagnosis website (http://dctd.cancer.gov/ResearchResources/ResearchResources-biomarkers.htm). A simplified version is also included in the Supporting Information (S1 Appendix).

γ-H2AX synthetic peptide and full-length H2AX recombinant protein were used for quantitative standard curve fit. A full length H2AX with a phosphoserine inserted at the site S139 was kindly provided by Dr. G. Wang from RANA Bioscience (Rockville, MD) to allow us to compare assay performance with synthetic peptide versus recombinant full length γ-H2AX as the γ-H2AX standard. This protein was prepared using an *in vitro* translation system and purified through a GST fusion at the N-terminal and His tag at the C-terminal [26].

### PAR ELISA assay

The validated PAR ELISA was performed as described [25], [27]. Briefly, PAR mouse monoclonal antibody (clone 10H) from Trevigen (Gaithersburg, MD) was added to each well of a 96-well white microtiter plate and incubated at 37°C for 2 hours. Wells were blocked with SuperBlock at 37°C for 1 hour. Pure PAR polymers (BioMol International, Plymouth Meeting, PA) were serially diluted in SuperBlock to reach concentrations ranging from 7.8 to 1000 pg PAR/mL to be used as standard controls. In duplicate or triplicate, either PAR standards or cell extracts were loaded per well and incubated at 4°C for 16 hours. PAR rabbit polyclonal antibody (Trevigen), diluted with 2% BSA in 1X PBS supplemented with normal mouse serum, was then added and incubated at 24°C for 2 hours. After incubation, HRP-conjugated affinity-purified goat anti-rabbit antibody (KPL, Gaithersburg, MD) was added. Freshly prepared SuperSignal ELISA Pico Chemiluminescent Substrate was added after 1 hour, and signal was immediately read on an Infinite M200 plate reader (Tecan). Relative light unit values minus background was plotted to generate standard curves.

### Microscope-based immunofluorescence assay (IFA)

For detection of γ-H2AX using microscopy, cells were fixed in 2% paraformaldehyde and suspended in 70% alcohol for cytospins, and tissues were fixed in formalin for paraffin block sections. Cytospin slides were prepared by centrifuging at 600 rpm for 10 minutes, with approximately 250,000 cells per EZ cytofunnel chamber (Fisher Scientific). For both cytospin and tissue slide staining of γH2AX, a biotinylated γ-H2AX monoclonal antibody was used (clone JBW301 from Millipore). Staining procedure and quantification method were previously described [12]. Briefly, γ-H2AX antibody attachment to nuclear DNA was reported using a conjugate reporter stain of either Alexa Fluor 555 (cytospin) or Alexa Fluor 488 (tissue). Slides were stained using a Bond-max Autostainer (Leica Microsystems, Wetzlar, Germany). Image capture was conducted on a Carl Zeiss 510 NLO or 710 NLO confocal microscope (cytospin) or on Leica DM500B and Nikon 90i fluorescent microscopes (tissue). A semi-automated custom macro capture script was operated in Image-Pro Plus v6. or v7.0 software (Media Cybernetics, Rockville, MD). Areas of interest to image were selected based on DAPI and Alexa Fluor 555 staining, excluding thick cellular areas with overlapping cells, areas with artifacts, or poor staining. Tissue and biopsy images were acquired by selecting regions of interest, excluding unacceptable areas that contained folds, scrapes, lack of tumor tissue, or excessive necrosis. Fields were imaged in the blue channel (Chroma A4 filter, BP 360/40) to measure DAPI-signal nuclear staining and then in the green channel (Chroma L5 filter, BP 480/40) to measure Alexa-Fluor labeled streptavidin bound to the biotin-conjugated γ-H2AX antibody. The average percentage of positively stained nuclear area from a minimum of 3 images of non-overlapping tumor cells was reported.

### Gene mutational status and “pattern comparison” to the ELISA outputs

Genetic variant data were accessed using the CellMiner database [28], [29]. The “amino acid changing” category includes only those variants defined as missense, nonsense, splice-sense, frameshift, read-through, or non-frameshift insertions or deletions that are projected to result in amino acid changes. “Protein function affecting” alterations include all of the alterations described for “amino acid changing”, plus several additional criteria. To be included as “protein function affecting”, the variant must be absent from both the ESP5400 (http://evs.gs.washington.edu/EVS) and the 1000 Genomes project [30], [31]. Also at least one of the following conditions must be true: 1) a SIFT score less than or equal to 0.05; 2) a Polyphen 2 score greater than or equal to 0.85; OR 3) the variant type must be defined as either nonsense, splice-sense, or frameshift. All of these are projected to affect protein function. Pattern comparisons of the ELISA and genetic variant data were done using the web-based application at “CellMiner \ NCI-60 Analysis Tools \ Pattern comparison” with cut-off at p<0.01 and genome analysis in the absence of multiple comparisons correction.

### Data analysis and statistics

For *H2AX* copy number correlation, data were obtained from genome microarrays accessible through CellMiner [23] and the Broad Institute CCLE data portal [24]. Regression analyses, Student's t-tests, and standard descriptive statistics were done using Microsoft Excel. For all lab assay data presented in graphs, each point represents the average of >2 replicates with standard deviations as indicated.

## Results

### Development of a sensitive ELISA-based assay

**Fig 1:**
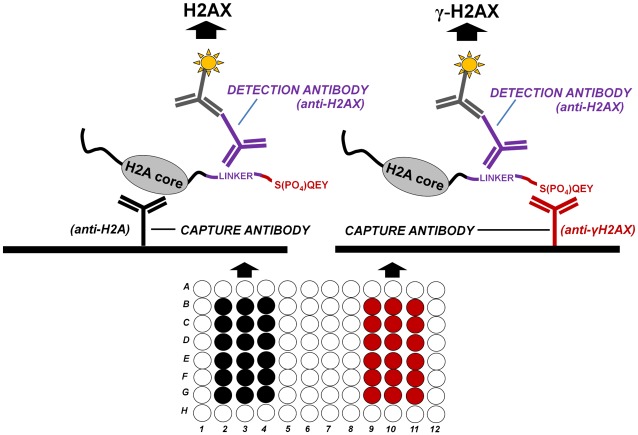
Schematic representation of the combined γ-H2AX and H2AX ELISA assay. The combined γ-H2AX/H2AX ELISA is performed by coating different wells of the same plate (or two different plates) with 2 different capture antibodies, each specific for a different part of H2AX: an anti-H2A antibody able to bind all histone H2A types, including H2AX, and γ-H2AX antibody able to bind only the phosphorylated form of H2AX (serine 139). Following incubation with the protein lysate, a detection antibody specific for the common linker region of H2AX is added in order to detect both H2AX and γ-H2AX. The H2AX antibody is then recognized by a HRP-labeled antibody in order to generate signal.

The sandwich ELISA method described here is depicted in Fig 1. Protein lysates are incubated with either an H2A antibody, capable of binding all histone H2A types including H2AX, or with a specific γ-H2AX antibody, able to capture only the phosphorylated form of H2AX. The γ-H2AX antibody (clone JBW301) has been described previously as being specific for an epitope containing phosphorylated serine 139 [32], [33]. Following incubation of the protein lysates with the two capture antibodies, the detection antibody is added. The detection antibody binds the H2AX linker sequence common to both unphosphorylated H2AX and H2AX phosphorylated on Ser139. This antibody is then recognized by a HRP-labeled detection antibody, providing a readout signal. The final result for a given sample is calculated by dividing the amount of γ-H2AX by the amount of total H2AX detected, expressed as the percent phosphorylated H2AX (see S1 Appendix for the detailed γ-H2AX/total H2AX ELISA protocol).

**Fig 2:**
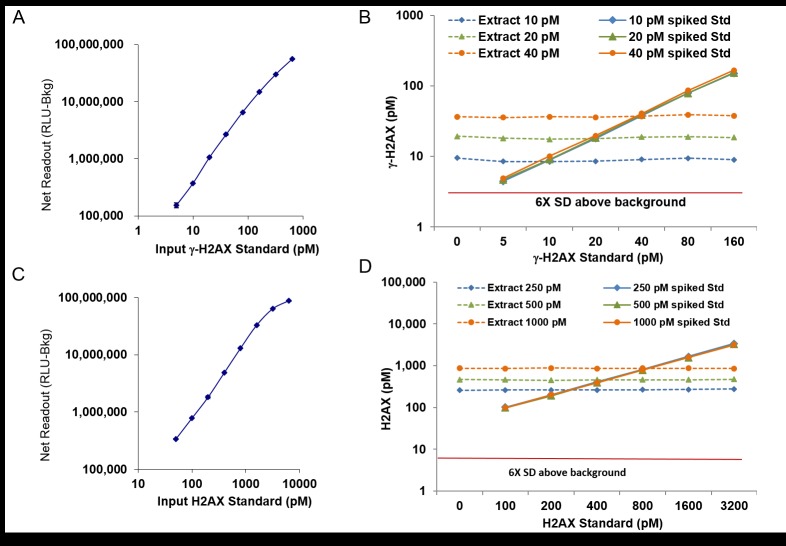
Consistency of standard curves for γ-H2AX and H2AX regardless of sample composition. **(A)** The net readout (Relative Light Unit minus Background [RLU-Bkg]) for various concentrations (pM) of the standard γ-H2AX peptide. The background (Bkg) with zero pM standard is about 500,000 RLU. **(B)** γ-H2AX measurements for an extract diluted to 10 pM, 20 pM, or 40 pM γ-H2AX and then spiked with increasing amounts of the standard γ-H2AX peptide. Solid lines represent the standard curves for the spiked samples after the baseline γ-H2AX concentrations of the extract dilutions were subtracted. Dashed lines represent the readout for each dilution of the extract after the amount of peptide added to each spiked sample was subtracted. **(C)** The net readout (RLU-Bkg) for various concentrations (pM) of the standard H2AX protein. **(D)** H2AX measurements for an extract diluted to 200 pM, 500 pM, or 1000 pM H2AX and then spiked with increasing amounts of the H2AX protein. Solid lines represent the standard curves for the spiked samples after the baseline H2AX concentrations of the extract dilutions were subtracted. Dashed lines represent the readout for each dilution of the extract after the amount of peptide added to each spiked sample was subtracted. For **(A-D)**, points represent average of 2 duplicates and error bars show standard deviation.

After developing the ELISA assay, we first investigated its sensitivity and specificity. As depicted in Fig 2, standard curves were performed using increasing concentrations of γ-H2AX (Fig 2A; dilution 5, 10, 20, 40, 80, 160, 320, 640 pM) and H2AX (Fig 2C; 50, 100, 200, 400, 800, 1600, 3200, 6400 pM) peptides, and the data exhibit linear relationships between the concentration of the antigens and the amount of signal detected. Furthermore, γ-H2AX and H2AX were clearly detectable at 5 pM and 50 pM respectively. To demonstrate the reliability of the assay, a well-characterized and quantified extract was diluted to 10 pM, 20 pM, or 40 pM γ-H2AX, and samples of each dilution were then spiked with increasing amounts of the standard γ-H2AX peptide. After the baseline γ-H2AX concentrations of the 10 pM, 20 pM, and 40 pM γ-H2AX extracts were subtracted, the standard curves for the spiked samples were consistent and accurately reflected the amount of peptide added (Fig 2B; solid lines). Similarly, after subtracting the amount of peptide added to each spiked sample, the assay readout for each sample remained stable and consistent with the input concentration of the extract (Fig 2B; dashed lines). These results indicate that the assay is precise and can accurately detect γ-H2AX levels across a wide range of sample dilutions, without interference from any sample component. The same validation was performed for total H2AX measurement and exhibited the same consistency across different sample compositions (Fig 2D). Similar experiments were also done to test for H2A interference, and there were no detectable effects on the standard curves when H2AX and γ-H2AX standards were spiked with 200, 800, 3200 and 26,500 pM H2A recombinant protein (data not shown).

It should be noted that a standard γ-H2AX peptide was used to construct the standard curve. However, we also evaluated assay performance with recombinant full length γ-H2AX and found equivalent affinity to γ-H2AX antibodies and similar quantitative detection (data not shown). We therefore suggest using the synthetic phosphorylated peptide due to its purity, stability, and ready availability.

### γ-H2AX ELISA assay readily detects DNA damage resulting from ionizing radiation

**Fig 3:**
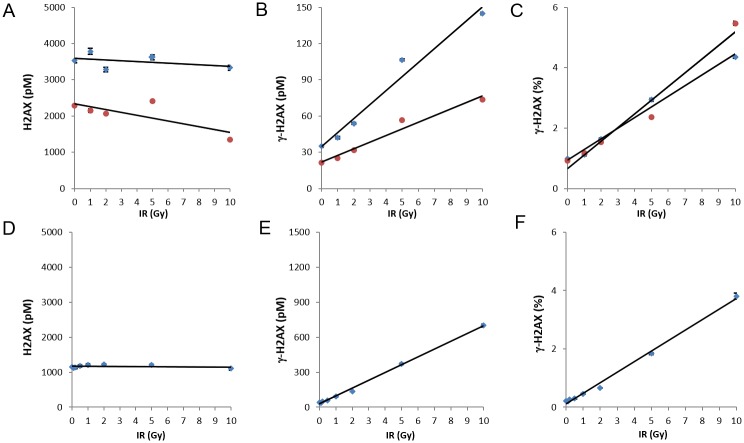
Detection of DNA damage caused by ionizing radiation. Cultures of the monocytic line THP1 (A-C) or of normal human fibroblasts (NHF; D-F) were exposed to various amounts of ionizing radiation up to 10 Gy, incubated for 30 minutes at 37°C, then solubilized at 1 x 10^7^ cells/mL. **(A-C)** For THP1, graphs show the absolute amounts of H2AX **(A)** and γ-H2AX **(B)** from the ELISA at two extract dilutions, 1:25 (blue diamonds) and 1:50 (red circles), as well as the resulting values for % γ-H2AX **(C),** independent of extract dilution in the assay. Error bars show standard deviation. The linear best-fit trendline is also shown. **(D-F)** For NHF, graphs show the absolute amounts of H2AX at a dilution of 1:50 **(D)** and γ-H2AX at a dilution of 1:3 **(E)**, as well as the resulting values for % γ-H2AX **(F)**. For example at 10 Gy, total H2AX = 1000(value) x 50(dilution) = 50,000 pM/10^7^ cells/mL. γ-H2AX = 600(value) x 3(dilution) = 1800 pM/10^7^ cells/mL. %γ-H2AX = 1800/50,000 = 3.6%. For **(A-F)**, points represent average of 2 duplicates and error bars show standard deviation.

Since γ-H2AX formation has been reported to be proportional to the amount of ionizing radiation-induced DNA DSBs [8], we first examined the utility of the γ-H2AX ELISA using ionizing radiation (IR) exposure. The first cell type studied was THP1, a transformed monocytic line. Absolute amounts of total H2AX (Fig 3A) and γ-H2AX (Fig 3B) are shown for two dilutions of THP1 (1:25 and 1:50 shown in blue and red data points, respectively) after exposure to various doses of radiation ranging from 1 to 10 Gy. As expected, the ELISA demonstrated a dose-dependent increase in γ-H2AX, indicating the presence of DSBs (Fig 3B). While the absolute values are dependent on the amount of extract loaded, the final % γ-H2AX values are not, demonstrating the robustness of the assay even with variability in extract concentrations (Fig 3C). For comparison, normal human fibroblasts (NHF) were also examined. There was an increase in % γ-H2AX with increasing IR exposure in the NHF cultures, similar to that observed in the THP1 experiments (Fig 3D–3F).

These ELISA results support the previously described observation that tumor lines often have basal % γ-H2AX levels higher than those in normal cells (Fig 3C and 3F; 1% for THP1 vs 0.2% for NHF) [3]. The increase in % γ-H2AX that occurs in THP1 in response to IR-induced DNA damage is therefore less striking; for NHFs, the % γ-H2AX value after 1 Gy IR is over twice that of the basal % γ-H2AX, while for THP1, it is only about 20% above the basal value. This difference in baseline % γ-H2AX expression emphasizes the need for a γ-H2AX assay that is able to measure a wide range of values and is sensitive enough to detect subtle changes in % γ-H2AX levels.

### Detection of drug-induced DNA damage with the γ-H2AX ELISA

**Fig 4:**
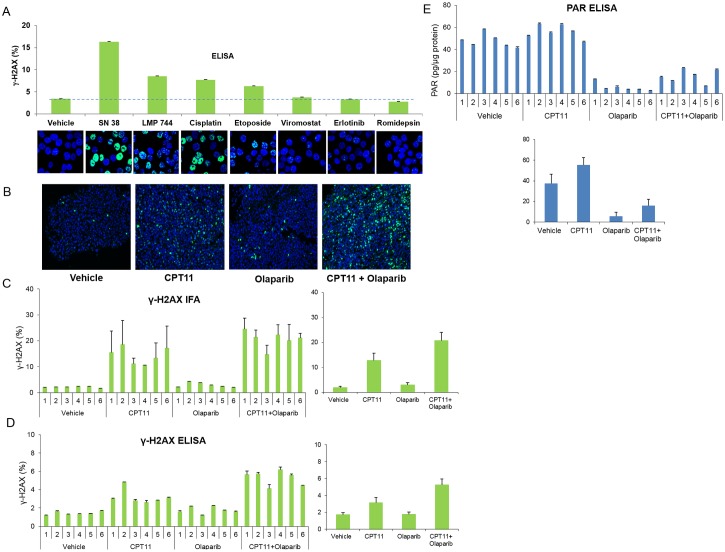
Comparison of ELISA with other DNA damage assays. **(A)** HT29 cell cultures were treated with the indicated drugs for 1 hour (with the exception of cisplatin, with which cells were treated for 6 hours). Drugs were added to cultures at the following concentrations: SN38, vorinostat, erlotinib, and romidepsin at 1 μM; LMP744 at 10 μM; and cisplatin and etoposide at 100 μM. Samples were then divided into two aliquots; one was prepared for microscopy and stained for γ-H2AX (green) and DNA (blue; DAPI) and the other was extracted and analyzed by ELISA. The % γ-H2AX values from the ELISA assay are shown in the bar graph above the stained IFA images. The dashed horizontal line indicates the value for vehicle. **(B)** Representative microscopy images from A375 xenograft mice treated with individual drugs or drug combination as indicated. A fragment of each xenograft was fixed and processed for γ-H2AX immunofluorescent staining (IFA). γ-H2AX staining is green; nuclei are stained with DAPI blue. **(C-E)** Bar graphs showing quantification of γ-H2AX IFA staining **(C)**, % γ-H2AX ELISA readings **(D)**, and PAR ELISA readings **(E)** in xenograft tumors. Mice were treated as indicated on the bottom of each figure. Readings from individual mice are shown on the left (top for **E**) and averages of the treatment cohorts (n = 6 for all) are on the right (bottom for **E**).

Because the microscopy-based γ-H2AX assay is the most sensitive tool to measure DSB induction, we investigated how the ELISA assay compares with microscopy for the assessment of DNA damage. Fig 4 shows that the ELISA results correspond with the findings of a microscopy-based assay for the assessment of cell responses to DNA-damaging agents. Cultures of the human colorectal adenocarcinoma cell line HT29 were exposed to the indicated drugs for 1 hour (with the exception of cisplatin, to which cells were exposed for 6 hours) and then evaluated by microscopy and ELISA (Fig 4A). Images from the microscopy immunofluorescence assay (IFA) clearly exhibit an increase in γ-H2AX levels after treatment with DNA-damaging drugs. While the cells treated with the vehicle or with the non-DNA-damaging agents vorinostat, erlotinib, or romidepsin [34–36] exhibit little, if any, difference in staining, the other drug treatments induced detectible increases in staining for γ-H2AX (Fig 4A, bottom panel). This result is consistent with the reported DNA-damaging effects of cisplatin, which causes DNA crosslinking, and of SN38, LMP744, and etoposide, which are topoisomerase inhibitors [37–40]. Importantly, values for % γ-H2AX measured in duplicate samples by the γ-H2AX ELISA demonstrate a trend similar to the observed immunofluorescent staining in the various treatment conditions, providing further support that the ELISA yields useful information regarding DNA damage levels (Fig 4A, top panel).

While *in vitro* experiments demonstrate that the % γ-H2AX ELISA provides accurate results for cell line extracts, a more common experimental objective is the analysis of protein levels in solid tissues such as tumors. We treated A375 melanoma xenograft mice with the DNA-damaging topoisomerase I (Top1) inhibitor CPT11 (irinotecan), the poly(ADP-ribose) polymerase (PARP) inhibitor olaparib, or a combination of both drugs. This combination was tested based on the findings of previous preclinical studies that have evaluated the effects of combining PARP and Top1 inhibitors. By blocking the activity of PARP enzymes, PARP inhibitors prevent the repair of DSBs, enhancing the cytotoxicity of DNA-damaging Top1 inhibitors [41], [42]. Mice were sacrificed within 24 hours of treatment, and tumor tissue was analyzed for levels of γ-H2AX, first by using the well-established IFA (Fig 4B and 4C). Fig 4B shows representative images of a xenograft tissue section for each treatment, with the fraction of nuclear area with positive staining quantified in Fig 4C (left, individual mice; right, cohort averages and standard deviations). There were minimal increases in γ-H2AX staining with olaparib treatment but dramatic increases with CPT11 compared to controls, results consistent with the previously described effects of these agents [43], [44]. An even more striking increase in γ-H2AX staining was observed after dual treatment with CPT11 and olaparib (p<0.005 for combination group versus CPT11-treated group), indicating synergistic induction and/or persistence of DNA breaks from the drug combination *in vivo*. The ELISA was then used to assess % γ-H2AX expression in the same tissues (Fig 4D; left, individual mice; right, cohort averages and standard deviations). The readouts demonstrate increases in γ-H2AX with CPT11 and with combination treatment, changes comparable to those seen with IFA.

The same xenograft samples prepared for the % γ-H2AX ELISA were also used in a separate ELISA measuring PAR levels, and as expected, inhibition of PARP (indicated by the decrease in PAR) was observed with olaparib and not with CPT11 (Fig 4E; top, individual mice; bottom, cohort averages and standard deviations) [35]. The PAR ELISA serves as a control readout, demonstrating the ability to obtain reliable results using the same protein extract for multiple assays. This major advantage of the γ-H2AX ELISA, as opposed to microscopy-based approaches, makes it possible to measure downstream targets in the DNA damage response pathway in the same cells and thus obtain a more comprehensive view of the signaling pathways that are activated in response to DSBs.

### Characterization of H2AX levels across NCI-60 cell lines

**Fig 5:**
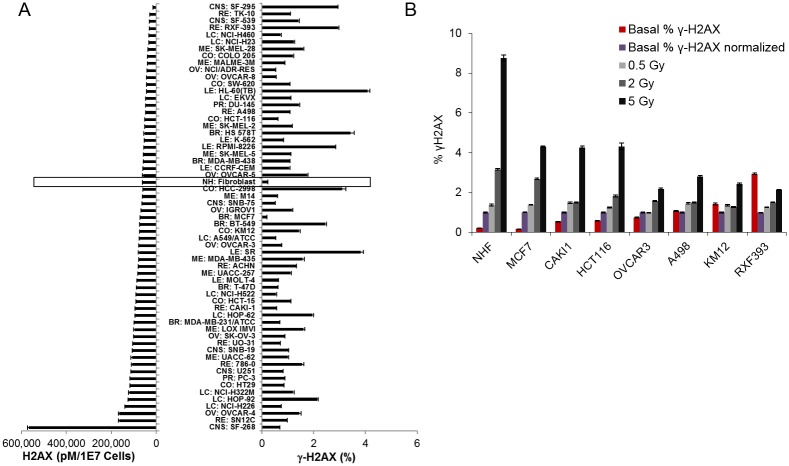
Studies of the NCI-60 cell databank using the combined % γ-H2AX ELISA. **(A)** Cell lines from the NCI-60 cell bank aligned in order of increasing total H2AX amounts per cell (left). The basal % γ-H2AX in unstressed cells for each cell line is shown on the right. Data from normal human fibroblasts (NHFs) is boxed. **(B)** Selected lines were arranged according to their basal % γ-H2AX values (red bars). Light gray, dark gray, and black bars represent the fold change in % γ-H2AX after 0.5, 2, and 5 Gy radiation, respectively, relative to the basal value (purple bars).

The ELISA is a promising tool for the high-throughput screening used in both drug discovery and patient sample analysis. To explore this potential, we utilized the γ-H2AX ELISA assay to simultaneously analyze multiple extracts from the NCI-60 cancer cell line panel. The NCI-60 panel contains 60 tumor cell lines representing breast, colon, lung, brain, ovary, prostate, and kidney cancer, as well as leukemia and melanoma [45]. The ELISA showed that, with the exception of the glioblastoma cell line SF268, total H2AX values varied from approximately 20 to 180 pM per 1 x 10^4^ cells/μL (Fig 5A, left hand panel). Interestingly, SF268 contains over 500 pM H2AX, due to a six-fold amplification of the H2AX gene and surrounding loci. NHFs are included in Fig 5 for comparison and exhibit a total H2AX value in the middle of the range (approximately 100 pM per 1 x 10^4^ cells/μL). Such a wide range in H2AX protein expression corresponds to the variability in H2AFX gene copy and/or transcript number that has been observed in tumor cells (S1A and S1B Fig) and emphasizes the importance of normalizing γ-H2AX measurements to the total amount of H2AX in the cell for accurate comparisons across cell types.

The NCI-60 cell lines also show a wide variety of basal % γ-H2AX values (Fig 5A, right hand panel), from a low of 0.2% found in NHFs and the breast cancer cell line MCF7 to greater than 4% observed in the acute myeloid leukemia cell line HL-60(TB). These basal measurements are from cell lines cultured under the conditions recommended for optimal viability and otherwise unperturbed. It is clear that the basal % phosphorylated H2AX is independent of the total H2AX content in the NCI-60 cell lines. As with the THP1 experiments, measurements of the NCI-60 cell lines demonstrated elevated endogenous γ-H2AX levels in cancer cells compared to normal cells, supporting the hypothesis that increased DNA damage is a hallmark of genomic instability in cancer development.

Given the wide variety of basal % γ-H2AX values, we investigated how the % γ-H2AX at baseline affects the DNA damage response to IR. Fig 5B shows that NHFs exhibited a robust dose-dependent γ-H2AX response to IR, as expected for normal cells, while among the tumor lines, the relative response became less robust as the basal % γ-H2AX increased. This observation highlights the high variability in response to IR among tumor cell lines, which in turn raises questions about the linkage between γ-H2AX levels and cellular response to DNA breakage.

### γ-H2AX levels across cell lines correlate with mutational load

The lack of a strong response to IR in some tumor cell lines that have high basal levels of % γ-H2AX may indicate that these lines have mutations that impair the response to DNA damage, and baseline expression of γ-H2AX may therefore be useful as a surrogate marker for defects in the DNA damage response pathway. In fact, it is possible to address this question because the NCI-60 panel has been extensively characterized, including complete exome sequencing of all 60 lines [29] [46]. The three ELISA outputs—total H2AX, γ-H2AX, and % γ-H2AX—were correlated with various characteristics of the NCI-60. The “Pattern Comparison” web-application found significant positive correlations within two categories of mutations present in the NCI-60 panel, the 12,706 “amino acid changed” and the 9,143 “protein function altered” genetic variants derived from exome sequencing of the NCI-60 (Table 1). Among the three inputs, the % γ-H2AX input was found to have significant positive correlations to 959, or over 10%, of the 9,143 “protein function altered” gene patterns. These protein function-affecting gene patterns provide an indication of the mutational status of the cancer cell lines in their untreated state, and the findings suggest that increased % γ-H2AX values correspond to high mutational burden. No significant correlation was found when total mutations were analyzed (data not shown), likely because the majority of mutations are neutral, without changing amino acid or affecting protein function.

**Table 1:** Correlations between mutational load and H2AX measurements in the NCI-60 cell bank.

		Amino acid changed		Protein function altered	
	Correlation direction	Number of genes	Percentage of data set	Number of genes	Percentage of data set
Total H2AX	+	241	1.90%	53	0.58%
	-	0	0%	0	0%
γ-H2AX	+	190	1.50%	60	0.66%
	-	0	0%	0	0%
% γ-H2AX	+	725	5.71%	959	10.49%
	-	0	0%	0	0%
Genes in data set		12,706		9,143	

The γ-H2AX, total H2AX, and % γ-H2AX values were compared to the “amino acid changed” and the “protein function altered” genetic variants derived from exome sequencing of the NCI-60 cell lines using the CellMiner “Pattern comparison” program. Correlation direction, number of genes, and percentage of data set are shown.

These results may indicate that the basal % γ-H2AX values have a physiological basis and are not simply due to environmental factors. While the other two inputs also had significant correlations, the results were much less striking (Table 1), with the ratio of γ-H2AX to total H2AX providing a 17-fold increase in signal strength compared to each alone. If future studies continue to support a correlation between % γ-H2AX and protein function-affecting gene mutations, % γ-H2AX measured by the sandwich ELISA method could potentially be explored as an indicator of tumor mutational load.

## Discussion

We have described here the utility of a novel and sensitive ELISA for the quantification of γ-H2AX levels relative to total H2AX expression in cells and tissues. This assay readily detects DNA damage induced by ionizing radiation or DNA-damaging agents and provides precise readouts regardless of sample composition. Compared to traditional microscopy-based approaches, the ELISA is quicker (can be performed within one working day), better suited to high-throughput screening, and has an internal control (total H2AX) for reliable measurements. The inclusion of a measurement for total H2AX helps ensure that any observed increases in γ-H2AX levels are indeed due to DNA damage and not caused by differences in baseline H2AX expression or non-uniformities in sample preparation. This control for variability across samples and cell types represents a substantial improvement over available assays for γ-H2AX alone.

When developing the γ-H2AX/total H2AX sandwich ELISA assay, we recognized that one of the difficulties in performing quantitative H2AX assays is the high background that results from the cross-reactivity of antibodies and highly charged histone proteins. We compared various diluents, including salmon sperm DNA, protamine sulfate, poly-L-lysine, and others, in order to improve signal-to-noise ratio and optimize the linear dynamics. We found that protamine suppressed background signal, improved linear response, and had the best recovery in our spiked sample experiment. Therefore, the H2AX ELISA described here includes a step in which protamine is loaded on the plate prior to the samples.

Importantly, extracts prepared for the γ-H2AX sandwich ELISA assay can be used for other ELISAs, Western blots, and other methods of protein analysis. This flexibility makes it possible to investigate the presence of other DNA damage response and apoptotic markers in the same samples, allowing investigators to assess downstream targets and pathways and perform more comprehensive molecular studies. However, when this capability is not required, a simpler histone extraction can be used for the ELISA, in which cell lysates, whole cells, or small pieces of tissue are extracted with 0.4 M HCl or 0.2 M H_2_SO_4_. The supernatants can then be neutralized with Tris base [47], [48]. Alternatively, histones can be precipitated from the acid supernatants by the addition of 100% trichloroacetic acid to a final concentration of 25%. These techniques may be advantageous when cell number is low, a common experimental scenario when evaluating tumor biopsy samples.

Analysis of γ-H2AX in tumor biopsies is most typically done by immunofluorescent staining. As our data show, the ELISA produces findings regarding changes in γ-H2AX levels similar to those obtained from the IFA. However, the basal % γ-H2AX measurements are higher with the ELISA than with the IFA. This difference is due to the nature of the assays. In IFA, sensitivity settings of the color channels are adjusted to minimize the average basal reading during image manipulation; thus, the readouts obtained from IFA are relative, compared to the absolute values attained with the ELISA. In addition, the IFA is performed by analyzing several sections for each xenograft, while the ELISA result is an average of several replicates from the same extract for each xenograft. These differences in sampling account for the smaller standard deviations in the ELISA data. Therefore, there is less variability in the higher basal readings obtained by the ELISA, making the smaller differences seen between controls and experimental samples as reliable as those obtained with the IFA.

Another dissimilarity between the ELISA and IFA assays is that the IFA is able to detect murine γ-H2AX and H2AX, while the ELISA assay is specific for human proteins. This difference is presumably due to the activity of the ELISA detection antibody, which targets the H2AX linker sequence between the conserved core and SQ tail sequences. Mouse H2AX contains a double amino acid substitution (AV for SG) in this linker region, which may interfere with binding by the detection antibody. The specificity of the ELISA to detect only human H2AX is potentially advantageous, as mouse cells may infiltrate the human xenografts and interfere with experimental results. However, the overall correlation between the IFA and ELISA for measurement of γ-H2AX among 24 xenograft tumors tested was 93%, indicating that there is general agreement between the IFA and the novel ELISA assay in the measurement of γ-H2AX in animal model experiments.

We have established the ability of the γ-H2AX ELISA assay to detect DNA damage in xenograft tumors after administration of DNA-damaging agents. Specifically, treatment with topoisomerase I (Top1) inhibitor CPT11 resulted in induction of H2AX phosphorylation and formation of γ-H2AX. The increase in γ-H2AX compared to vehicle was even more significant when tumor-bearing mice were treated were a combination of CPT11 and the PARP inhibitor olaparib. This result is consistent with the findings of previous preclinical studies that have found synergistic effects when PARP and Top1 inhibitors are combined in cultured cells and animal models. In fact, combination therapy with PARP and Top1 inhibitors, including dual treatment with CPT11 and olaparib, has potential in human cancer treatment and is already being investigated in patients with various types of tumors and lymphomas [49–53]. The γ-H2AX ELISA assay represents a useful tool that could improve the clinical study of these drugs and their effects on cancer cells.

The experiments presented here also demonstrate the potential of the γ-H2AX ELISA to drive greater understanding of the mechanisms involved in the response to DNA damage. We have used the assay to correlate baseline γ-H2AX levels with the robustness of DNA damage response, as well as to reveal a potential relationship between % γ-H2AX and tumor mutational load. These findings warrant further investigation and are just two examples of the novel studies that can be performed using the sandwich γ-H2AX ELISA method. Widespread use of this assay will allow for more comprehensive study of DNA damage responses in tumors, ultimately supporting the development of more effective DNA-damaging anti-cancer agents.

## Supporting information

S1 Fig Wide range of expression of *H2AX*. Plots showing the correlation between *H2AX* gene copy number and H2AX transcript number across **(A)** the 60 cell lines in the NCI-60 cancer cell bank and **(B)** the 1,008 cell lines in the Cancer Cell Line Encyclopedia (CCLE). Data were obtained from genome microarrays accessible through CellMiner [23] and the Broad Institute CCLE data portal [24]. X-axis represents Log2 intensity values for transcripts and Y-axis represents copy number.
(TIF)

S1 Appendix Laboratory protocols for the γ-H2AX and H2AX ELISA assays. Simplified protocols for the γ-H2AX and H2AX sandwich ELISA assays are provided. More detailed instructions can be found on the NCI Division of Cancer Treatment and Diagnosis website (http://dctd.cancer.gov/ResearchResources/ResearchResources-biomarkers.htm).
(DOCX)
