# Supplementary figures and images for: Phosphorylated fraction of H2AX as a measurement for DNA damage in cancer cells and potential applications of a novel assay
Source: PLoS One. 2017 Feb 3;12(2):e0171582. doi: 10.1371/journal.pone.0171582 (PMC5291513; doi:10.1371/journal.pone.0171582)

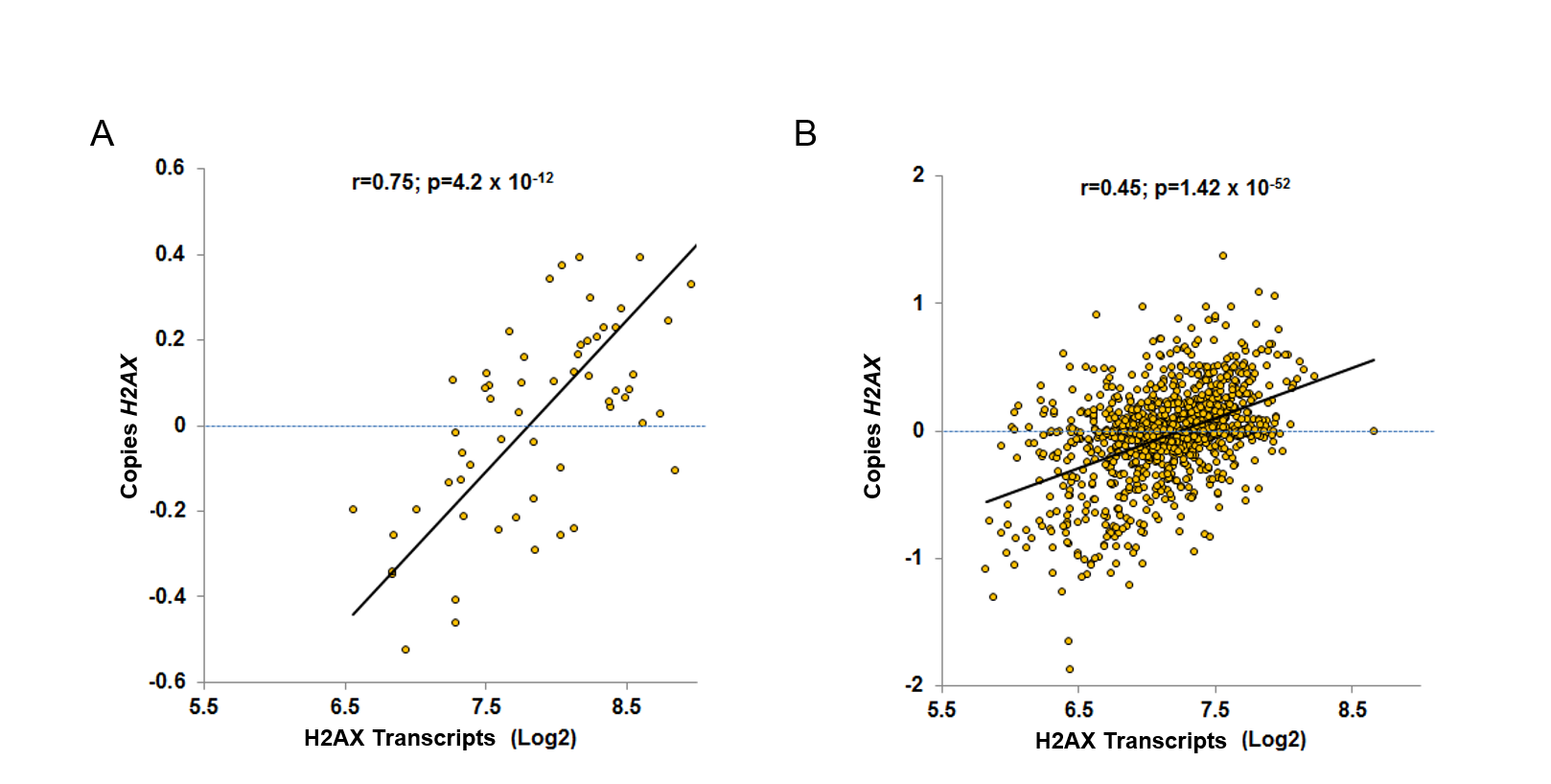

Supplement: S1 Fig — Plots showing the correlation between H2AX gene copy number and H2AX transcript number across (A) the 60 cell lines in the NCI-60 cancer cell bank and (B) the 1,008 cell lines in the Cancer Cell Line Encyclopedia (CCLE). Data were obtained from genome microarrays accessible through CellMiner [23] and the Broad Institute CCLE data portal [24]. X-axis represents Log2 intensity values for transcripts and Y-axis represents copy number. (TIF) [file pone.0171582.s001.tif]
