# Supplementary material for: Phosphorylated fraction of H2AX as a measurement for DNA damage in cancer cells and potential applications of a novel assay
Source: PLoS One. 2017 Feb 3;12(2):e0171582. doi: 10.1371/journal.pone.0171582 (PMC5291513; doi:10.1371/journal.pone.0171582)
Supplement: S1 Appendix — Simplified protocols for the γ-H2AX and H2AX sandwich ELISA assays are provided. More detailed instructions can be found on the NCI Division of Cancer Treatment and Diagnosis website (http://dctd.cancer.gov/ResearchResources/ResearchResources-biomarkers.htm). (DOCX) [file pone.0171582.s002.docx]

# S1 Appendix. Laboratory protocols for the γ-H2AX and H2AX ELISA assays.

## Protocol for γH2AX Enzyme Linked Immunosorbent Assay (ELISA)

1. MATERIALS and EQUIPMENT
   1. γH2AX peptide standard, lyophilized powder (custom-preparation from Invitrogen, synthetic peptide: AVLLPKKTSATVGPKAPSGGKKATQA[PS]QEY)
   2. Tumor Lysate Control (custom preparation prepared to target low, mid and high γH2AX ranges)
   3. Phospho-H2AX (Ser139) mouse monoclonal antibody, clone JBW301 (Millipore, Cat#: 05-636)
   4. Histone H2AX rabbit polyclonal antibody (Abcam, Cat#: ab10475)
   5. Goat anti-rabbit HRP-conjugated polyclonal antibody, 1 mg (KPL, Cat#: 074-15-061), Dilute to 1 mg/mL stock solution in HRP Stabilizer (KPL, Cat#: 54-15-01)
   6. SuperSignal ELISA Pico Chemiluminescent Substrate (Thermo Scientific Pierce, Cat#: 37070)
   7. Acetate plate sealers (Thermo Scientific Pierce, Cat #: 3501)
   8. Reacti-Bind White Opaque 96-well Plate (Thermo Scientific Pierce, Cat#: 15042)
   9. Pipettors (200-1000 µL, 50-200 µL, and 2-20 μL) and tips
   10. Multichannel pipettors (50-300 µL, 5-50 µL) and tips
   11. Reagent reservoirs (Fisher Scientific, Cat#: 21-381-27C)
   12. 1.5-mL Sarstedt tubes (Sarstedt, Cat#: 72.692.005)
   13. 15-mL polypropylene tubes (e.g., Fisher Scientific, Cat#: 14-959-49B)
   14. 50-mL polypropylene tubes (e.g., Becton Dickinson, Cat#: 352098)
   15. Ice bucket
   16. Carbonate-bicarbonate buffer capsules, pH 9.6 (e.g., Sigma-Aldrich, Cat#: C3041-50CAP)
   17. Tween 20 (e.g., Sigma-Aldrich, Cat#: P 1379)
   18. 10X Phosphate Buffered Saline, pH 7.2 (PBS; e.g., Invitrogen, Cat#: 70013-073)
   19. SuperBlock (TBS) Blocking Buffer (Thermo Scientific Pierce, Cat#: 37535)
   20. Albumin, bovine serum (BSA; e.g., Sigma-Aldrich, Cat#: A 7030)
   21. Mouse serum (e.g., Sigma-Aldrich, Cat#: M 5905)
   22. Cell Extraction Buffer (CEB; Invitrogen, Cat#: FNN0011)
   23. 20% sodium dodecyl sulfate (SDS; e.g., Sigma-Aldrich, Cat#: 05030-500ML-F)
   24. Sorvall Fresco microcentrifuge (Fisher Scientific)
   25. Vortex Genie 2 (Daigger, Cat#: 3030A)
   26. Dry, heated incubator able to maintain 37°C ± 3°C
   27. Dry, heated incubator able to maintain 25°C ± 3°C
   28. Infinite® 200 Microplate Reader (Tecan US)
   29. BioTek ELx405 Select Microplate Washer (BioTek Instruments)
   30. -80°C freezer
   31. 4ºC refrigerator

1. Example ELISA Plate Configuration

|  | **1** | **2** | **3** | **4** | **5** | | **6** | **7** | | **8** | **9** | | **10** | | **11** | | **12** | |
| --- | --- | --- | --- | --- | --- | --- | --- | --- | --- | --- | --- | --- | --- | --- | --- | --- | --- | --- |
| **A** | 1X PBS-2% BSA Only | | | | | | 5 pM | | | 1X PBS-2% BSA Only | | | | | | | | |
| **B** | High-C | S1 | S3 | S5 | S7 | | 10 pM | | | S9 | | S11 | | S13 | | S15 | | Low-C |
| **C** |  |  |  |  |  |  | 20 pM | | |  |  |  |  |  |  |  |  |  |
| **D** | Mid-C |  |  |  |  |  | 40 pM | | |  |  |  |  |  |  |  |  | Mid-C |
| **E** |  | S2 | S4 | S6 | S8 | | 80 pM | | | S10 | | S12 | | S14 | | S16 | |  |
| **F** | Low-C |  |  |  |  |  | 160 pM | | |  |  |  |  |  |  |  |  | High-C |
| **G** |  |  |  |  |  |  | 320 pM | | |  |  |  |  |  |  |  |  |  |
| **H** | 1X PBS-2% BSA Only | | | | | | 640 pM | | | 1X PBS-2% BSA Only | | | | | | | | |
|  |  |  | | | |  | | |  | | | | | | | | |  |
|  | Control Samples | Unknown Samples, Triplicate | | | | γH2AX Peptide Standards,  Duplicate | | | Unknown Samples, Triplicate | | | | | | | | | Control Samples |

S1 through S16 are unknown sample (S) wells in triplicate.

1. Sample Lysate Preparation

Unknown samples should be processed following SOP340520-Biopsy Specimen Processing for the DCTD Immunoassay (<http://dctd.cancer.gov/ResearchResources/ResearchResources-biomarkers.htm>) with the following **modifications**:

- 1. SOP340520 sample lysis references use of an 18-g needle biopsy. For preclinical sample processing, a similar tissue piece would weigh approximately 10 mg.
  2. In SOP340520, add 1 tablet PhosSTOP (Roche Applied Science, Cat#: 04906837001) per 10 mL Cell Extraction Buffer (CEB) for all steps requiring “CEB with PIs.”

These lysis procedures are compatible with the ELISA protocol outlined in the following operating procedures.

1. elisa pROTOCOL
   1. **Key Reagents**

It is recommended to record the lot numbers, stock reagent concentration, and expiration dates for the Key Reagents in the lab record.

- - 1. **γH2AX Peptide Standard**: Prepare as a 20680 pM stock solution. Aliquot in sufficient volumes for one 96-well plate; store at -80°C for ≤ 1 y.
    2. **Tumor Lysate Control**: Store at -80°C for up to 1 y. Lysates prepared to target Low, Medium and High γH2AX ranges.
    3. **γH2AX mAb**: Stock solution qualified from the manufacturer. Dilutions needed for assay performance for specific lot numbers of antibody will need to be determined by the assay site and should be matched to the pAb. Aliquot in sufficient volumes for one 96-well plate; store at ‑20°C for ≤ 6 mo.
    4. **H2AX pAb**: Stock solution qualified from the manufacturer. Dilutions needed for assay performance for specific lot numbers of antibody will need to be determined by the assay site and should be matched to the mAb Aliquot in sufficient volumes for one 96-well plate; store at ‑20°C for ≤ 6 mo.
    5. **Goat Anti-Rabbit HRP-Conjugated pAb**: Prepare a 1 mg/mL stock solution in HRP Stabilizer. Aliquot in sufficient volumes for three 96-well plates; store at 2°C to 8°C for ≤ 1 y.
    6. **Chemiluminescent Substrate Solutions**: Stock solutions (Peroxide and Pico Luminol/Enhancer Solutions) qualified from the manufacturer. Store at room temperature (25°C ± 3°C) for ≤ 3 mo. Protect from light during storage and use.
    7. **Reacti-Bind White Opaque 96-well Plate**: Store at 25°C ± 3°C away from volatile chemicals.
  1. **Plate Map and Buffer Preparation**
     1. Based on the number of unknown samples to be analyzed, generate a Plate Map (example, Step 2.0) to define the location and replicates of unknown samples, tumor lysate controls, and γH2AX peptide standards. Samples from a single experiment should be analyzed on one 96-well plate, not split over two, to ensure consistent sample handling.
     2. Once the number of wells is known, determine the amount of reagents required for the assay.

**IMPORTANT: For all of the following wash and aspiration steps, do not let the wells dry out.**

- 1. **Plate Preparation**
     1. Prepare 11 mL **γH2AX mAb** **Coating Solution** at a final concentration of 4µg/mL in 0.1 M pH9.6 carbonate buffer for the assay. This is sufficient for one 96-well plate (preparing enough for 110 wells). Thaw antibody immediately prior to dilution; do not allow sitting for extended periods upon thawing.
        1. If more than one 96-well plate is to be coated, pool the aliquots and then dilute appropriately. This will ensure that all plates are exposed to identical coating antibody. Discard excess diluted antibody.
     2. Add 100 µL of the **γH2AX mAb** **Coating Solution** per well using a multichannel pipettor, cover the plate with an acetate sheet, and incubate at 37°C for 2 h.
        1. Alternatively, the plate can be incubated overnight at 2°C to 8°C.
     3. Following incubation with the **γH2AX mAb** **Coating Solution**, aspirate the plate using a plate washer (for the BioTek Plate Washer, use the *Aspirate* program). After aspiration, tap the plate on paper towels to remove any residual liquid.
     4. Add 250 µL of SuperBlock to each well. Cover the plate with an acetate sheet and incubate at 37°C for 1 to 1.5 h.
        1. After blocking, move plate to 25°C ± 3°C until washing step (Step 4.7.1).
        2. Once coated plates have been blocked they can be stored at 2°C to 8°C for up to one week; do not let wells dry out.
  2. **Prepare Working Dilutions of Unknown Tumor Lysates**
     1. Stock lysates and protein concentration for tumors are prepared according to SOP340520.
     2. Tumor stock lysates with total protein concentration of < **0.2 μg/µL** should not be used in the γH2AX Immunoassay.
     3. Based on the protein measurements for the **stock tumor lysate** (μg/μL), prepare the **Working Lysates** in γH2AX assay diluent on ice for use in the γH2AX Immunoassay. Do not pipette less than 5 µL. If the calculations below yield volumes of stock lysate less than 5 µL, prepare sufficient volume of a 1:5 pre-dilution of the lysate before proceeding.
        1. For unknown stock lysates with stock protein concentrations ≥ **0.2 µg/µL:**
- Prepare 85 µL of a 0.2 µg/µL **Working Lysate** as follows:

| 0.2 µg/µL  **Working Lysate** | * | 85 µL | = | XX μL Vol. Stock Lysate to use |
| --- | --- | --- | --- | --- |
| XXX µg/µL  Conc. Stock Lysate | | |  |  |
|  |  |  |  | |

- In a labeled 1.5 mL tube, add sufficient γH2AX assay diluent to the calculated volume of stock lysate needed to bring total volume to 85 µL.
  1. **Preparation of Unknown Tumor or PBMC Lysate Samples**
     1. Place all unknown samples to be assayed on ice. Each unknown tumor lysate will take up 3 sample spots (e.g., S1, S2, and S3).
     2. Tumor lysate samples
        1. For unknown **Working Lysates** with protein concentrations of **0.2 µg/µL**,
        - Perform the following calculation to prepare 3 different lysate dilutions (2, 1, or 0.5 µg/well) in 100 μL total volume. For each **Diluted Lysate**, γH2AX assay diluent should be used to bring the total volume to 100 μL. This is sufficient volume to run each dilution in triplicate (plus 1 extra well). Clearly label all tubes with the sample number (e.g., S1, S2).
        - Record volume **Working Lysate** and γH2AX assay diluent used to prepare each **Diluted Lysate** in the lab record.

| (2, 1, or 0.5) µg/well  **Diluted Lysate** | * | 4 wells | = | (40, 20 or 10) μL **Working Lysate** |
| --- | --- | --- | --- | --- |
| 0.2 µg/µL  **Working Lysate** |  |  |  |  |
|  |  |  |  | |

- - - 1. Lysates will be diluted an additional 3‑fold with 1X PBS-2% BSA once loaded into the 96-well plate.
      2. Keep samples on ice until use. Only aliquot enough of each unknown sample for the assay.
    1. PBMC lysate samples
       1. Stock lysates for PBMCs (1 x 10^7^ cells/mL) are prepared according to SOP340506.
       2. Place 100 μL of the stock lysate into a clearly labeled 1.5-mL tube; this is enough for a triplicate well preparation (plus 1 extra well). Clearly label all tubes with the sample number (e.g., S7, S8). Alternatively, the original stock lysate can be directly used for loading. Make sure clearly label all sample tubes with the sample number (e.g., S7, S8).
       3. Lysates will be diluted an additional 3‑fold with 1X PBS-2% BSA once loaded into the 96-well plate yielding a relative load of 2.5 x 10^5^ cells/well.
       4. Keep all the samples on ice until use.
  1. **Preparation of γH2AX Peptide Standards and Tumor Lysate Controls**
     1. Preparation of γH2AX peptide standards; run in duplicate
        1. For one 96-well plate, retrieve a γH2AX peptide standard stock tube (20680 pM) from the -80°C freezer and thaw on ice. Vortex and mix by inverting 5-8 times before use. Label eight 1.5-mL Sarstedt tubes, numbered 1 through 8, for the γH2AX peptide standards. Prepare a 9^th^ tube as the blank.
        2. Prepare the γH2AX peptide standards by serial dilution with final concentrations ranging from 1920 to 15 pM in 1X PBS-2% BSA.
        3. Standards will be diluted an additional 3‑fold when added to the 96-well plate to generate a reference curve ranging from 640 to 5 pM γH2AX peptide standard.
        4. Keep standards on ice until use. Only make enough standards for the assay and discard any excess.
     2. Preparation of tumor lysate controls; run twice on plate in duplicate
        1. For one 96-well plate, retrieve one of each High-C, Mid-C and Low-C tumor lysate control vial from the -80°C freezer and thaw on ice. Controls are provided at a concentration ready for use in the assay and no further dilution is required. Vortex and mix by inverting 5-8 times before use.
        2. Controls will be diluted an additional 3‑fold with 1X PBS-2% BSA once loaded into the 96-well plate.
        3. Keep controls on ice until use. Controls will be diluted 3‑fold with 1X PBS-2% BSA once loaded into the 96-well plate.
  2. **γH2AX Protein Capture**
     1. Following incubation with SuperBlock, the plates are aspirated and washed once with 350 µL of 1X PBS-0.1% Tween using a plate washer.

For the BioTek Microplate Washer, the settings are:

| **METHOD** |  |
| --- | --- |
| Number of Cycles: | **1** |
| Soak/Shake: | **No** |
| **DISPENSE** |  |
| Dispense Volume: | **350 µL/well** |
| Dispense Flow Rate: | **06** |
| Dispense Height: | **120 (15.240 mm)** |
| Horizontal DISP POS: | **00 (0.000 mm)** |
| Bottom Wash First: | **No** |
| Prime Before Start: | **No** |
| **ASPIRATE** |  |
| Aspirate Height: | **031 (3.937 mm)** |
| Horizontal ASPR POS: | **-20 (-0.914 mm)** |
| Aspiration Rate: | **05 (6.4 mm/sec)** |
| Aspirate Delay: | **1000 MSec** |
| Crosswise ASPIR: | **No** |
| Final Aspiration: | **Yes** |
| Final Aspirate Delay: | **1000 MSec** |
|  |  |

- - 1. After the wash, tap the plate on paper towels to remove residual buffer. Proceed immediately to the next step; do not allow the plate to dry out.
    2. Immediately, add 50 µL of 1X PBS-2% BSA to each well using a multichannel pipettor. Each well will hold a final volume of 75 μL after sample addition.
    3. Use the Plate Map as a guide to set up the 96-well plate for incubation with unknown samples, γH2AX peptide standards, and tumor cell controls. Pipette reagents in the following order; **do not deviate** from order of addition:

| **Order** | **Sample/Reagent and Volume** |
| --- | --- |
| 1 | 25 μL of specified concentrations of γH2AX peptide standards into designated duplicate wells. Load the lowest concentration first. |
| 2 | 25 μL of each unknown sample into designated triplicate wells. |
| 3 | 25 μL each of assay control (Low-C, Mid-C, and High-C) into both sets of designated duplicate wells. |
| 4 | 25 μL of additional 1X PBS-2% BSA into each of the Background wells. |
|  |  |

- - 1. Cover the plate with an acetate sheet and incubate at 2°C to 8°C for 18 ± 2 h.
  1. **γH2AX Detection (next day)**
     1. Prepare a sufficient amount of the H2AX rabbit detection pAb 15 min before washing the plate (next step) that has been incubating with samples.
        1. Prepare 11 mL H2AX rabbit pAb working solution at a final concentration of 2µg/mL in 1X PBS-2% BSA supplemented with 1µL/mL mouse serum. This is sufficient for one 96-well plate (preparing enough for 110 wells). Thaw antibody immediately prior to dilution; do not allow sitting for extended periods upon thawing.
        2. Allow the prepared H2AX rabbit pAb to incubate for 15 min at 25°C ± 3°C.
     2. After the 18-h incubation is complete, aspirate and wash the wells 4 times with 350 µL of 1X PBS-0.1% Tween (same wash program as Step 4.7.1, except run for 4 cycles).
     3. After the wash, tap the plate on paper towels to remove residual Wash Buffer. Proceed immediately to the next step; do not allow the plate to dry out.
     4. Add 100 µL of the H2AX rabbit pAb working solution per well using a multichannel pipettor, cover the plate with an acetate sheet, and incubate for 2 to 2.5 h at 25°C ± 3°C. Discard residual working solution.
     5. 15 min before the incubation with the H2AX rabbit pAb is complete, prepare a sufficient amount of HRP conjugate for the assay.
        1. Prepare 11 mL HRP conjugate working solution at a final concentration of 1 µg/mL diluted in 1X PBS-2% BSA supplemented with 1µL/mL mouse serum. This is sufficient for one 96-well plate (preparing enough for 110 wells).
        2. Allow the prepared HRP conjugate to incubate in the dark at 25°C ± 3°C for 15 min.
     6. After the 2 to 2.5 h incubation with the H2AX rabbit pAb is complete, aspirate and wash the wells 4 times with 350 µL of 1X PBS-0.1% Tween (same wash program as Step 4.7.1, except run for 4 cycles). Tap plate on paper towels to remove residual liquid and proceed immediately to the next step.
     7. Add 100 µL of the HRP conjugate working solution per well using a multichannel pipettor. Cover the plate with an acetate sheet and incubate in the dark for 1 to 1.5 h at 25°C ± 3°C. Discard residual working solution.
  2. **Signal Detection**

| Shaking duration: | **5 sec** |
| --- | --- |
| Mode: | **linear** |
| Amplitude: | **1 mm** |
| Attenuation: | **OD1** |
| Integration Time: | **100 ms** |
|  |  |

- - 1. Turn on the Tecan Infinite Plate Reader at least 30 min before use. For luminescence optical density readings, the plate reader should be set to the following reading parameters:
    2. Just before the HRP conjugate incubation is finished, prepare 11 mL SuperSignal ELISA Pico Chemiluminescent Substrate Solution (5.5mL of Pico Stable Peroxide and 5.5mL of Pico Luminol/Enhancer). This must be made up immediately before use, kept in the dark, and at a sufficient volume for the assay.
    3. After the 1 to 1.5 h HRP conjugate incubation is complete, aspirate and wash the wells 4 times with 350 µL of 1X PBS-0.1% Tween (same wash program as Step 4.7.1, except run for 4 cycles). Tap plate on a paper towel to remove excess buffer and proceed immediately to the next step.
    4. Add 100 µL of the freshly made Substrate Solution per well with a multichannel pipettor and avoid bright light.
    5. The first chemiluminescence reading should be within 2 min of substrate addition.
       1. If the signal is too high from the initial reading, wait 5 min and read the plate again at the same instrument setting. Continue reading until the RLU signal is on scale.
    6. Save the resulting readings as an Excel file.
    7. Use the RLU (relative light unit) values from the Excel file to generate the γH2AX standard curve.
    8. Use the standard curve to convert RLU readings of the unknowns to γH2AX readings in pM.

1. qUALITY cONTROL Recommendations
   1. **Background Well QC**
      1. Use the Plate Map for the identification of background wells that are to be used in QC determination. A total of 14 wells are used for background determination; the 4 corner wells and 2 adjacent to the high standard are not used for background level calculation.
      2. A ± 2 SD criterion is applied to the initial 14-well dataset to identify outliers.
         1. If a background well RLU value is ≥ 2 SD from the mean, delete that value from the background dataset cells.
      3. Once all wells that were ≥ 2 SD from the initial background dataset mean have been deleted, the %CV for the background wells must be < 20%.
         1. If the %CV for the background wells is < 20%, the assay passes QC, proceed to Step 5.2.
         2. If the %CV is ≥ 20%, the **Assay Fails QC**, do not continue with the analysis.
   2. **Standard Curve QC**

If at any point, the “**Assay Fails QC**”. Rerun the assay with fresh reagents.

- - 1. **Low Standard QC and LLQ Assignment:**
    - In order to use the 5 to 10 pM range of the standard curve, the mean RLU readout of the 5 pM standard must be ≥ 3 SD above the mean RLU readout of the background; this value is referred to as the LLQ-RLU.
    - If the 5 pM standard fails, then the mean RLU readout of the 10 pM standard must be ≥ 3 SD above the mean RLU readout of the background.
    - If the 10 pM standard also fails, the **Assay Fails QC**.
    - The lowest passing standard is assigned as the LLQ (pM) for the assay.
    1. **Signal-to-background (S/B) ratio QC and ULQ Assignment:**
    - The ratio for the lowest passing standard (5 or 10 pM) RLU readout to the mean RLU readout of the background must be ≥ 1.1. If not, the **Assay Fails QC**.
    - The ratio of the highest standard RLU readout (640 pM) to the mean RLU of the background must be ≥ 15. If not, the **Assay Fails QC**.
    - If the high standard passes QC, it is assigned as the ULQ (pM) for the assay.
  1. **Control Samples**
     1. The QC determination for the control samples should have the following criteria:
     - At least one control at each level (Low-, Mid-, and High-C) must have a CV of < 20% for the replicate wells.
     - At least one control at each level and at least 4 of 6 controls overall must fall within the defined γH2AX pM range provided for the **specific lot** of critical reagent.
     1. If any of these criteria are not met, the **Assay Fails QC**. State in the lab record the reason for assay failure. Rerun the assay with fresh reagents.
  2. **Unknown Sample Replicate QC and LLQ/ULQ QC**
     1. Triplicate repeats for each sample must have a CV < 20%.
     2. Review the average γH2AX levels and identify any values that are < LLQ or > ULQ.
        1. If a sample is > ULQ and there is sufficient sample volume, it can be re-run with fresh reagents at a 2-fold lower protein load/well. If a sample is < LLQ and there is sufficient sample volume, it can be re-run at a 2-fold higher protein load/well.

## Protocol for H2AX Enzyme Linked Immunosorbent Assay (ELISA)

1. MATERIALS and EQUIPMENT
   1. Total H2AX recombinant standard, lyophilized powder (Axxora, Cat#: ALX-201-176-M005)
   2. H2A monoclonal antibody, 4F10 (Novus, Cat#: H00008334-M01)
   3. H2AX rabbit polyclonal antibody (Abcam, Cat#: ab10475)
   4. Goat anti-rabbit HRP-conjugated polyclonal antibody, 1 mg (KPL, Cat#: 074-15-061). Dilute to 1 mg/mL stock solution in HRP Stabilizer (KPL, Cat#: 54-15-01)
   5. SuperSignal ELISA Pico Chemiluminescent Substrate (Thermo Scientific Pierce, Cat#: 37070)
   6. Acetate plate sealers (Thermo Scientific Pierce, Cat #: 3501)
   7. Reacti-Bind White Opaque 96-well Plate (Thermo Scientific Pierce, Cat#: 15042)
   8. Pipettors (200-1000 µL, 50-200 µL, and 2-20 μL) and tips
   9. Multichannel pipettors (50-300 µL, 5-50 µL) and tips
   10. Reagent reservoirs (Fisher Scientific, Cat#: 21-381-27C)
   11. 1.5-mL Sarstedt tubes (Sarstedt, Cat#: 72.692.005)
   12. 15-mL polypropylene tubes (e.g., Fisher Scientific, Cat#: 14-959-49B)
   13. 50-mL polypropylene tubes (e.g., Becton Dickinson, Cat#: 352098)
   14. Ice bucket
   15. Carbonate-bicarbonate buffer capsules, pH 9.6 (e.g., Sigma-Aldrich, Cat#: C3041-50CAP)
   16. Tween 20 (e.g., Sigma-Aldrich, Cat#: P 1379)
   17. 10X Phosphate Buffered Saline, pH 7.2 (PBS; e.g., Invitrogen, Cat#: 70013-073)
   18. SuperBlock (TBS) Blocking Buffer (Thermo Scientific Pierce, Cat#: 37535)
   19. Albumin, bovine serum (BSA; e.g., Sigma-Aldrich, Cat#: A 7030)
   20. Mouse serum (e.g., Sigma-Aldrich, Cat#: M 5905)
   21. Cell Extraction Buffer (CEB; Invitrogen, Cat#: FNN0011)
   22. 20% sodium dodecyl sulfate (SDS; e.g., Sigma-Aldrich, Cat#: 05030-500ML-F)
   23. Sorvall Fresco microcentrifuge (Fisher Scientific)
   24. Vortex Genie 2 (Daigger, Cat#: 3030A)
   25. Dry, heated incubator able to maintain 37°C ± 3°C
   26. Dry, heated incubator able to maintain 25°C ± 3°C
   27. Infinite® 200 Microplate Reader (Tecan US)
   28. BioTek ELx405 Select Microplate Washer (BioTek Instruments)
   29. -80°C freezer
   30. 4ºC refrigerator

1. Example ELISA Plate Configuration

|  | **1** | **2** | **3** | **4** | **5** | | **6** | **7** | | **8** | **9** | | **10** | | **11** | | **12** | |
| --- | --- | --- | --- | --- | --- | --- | --- | --- | --- | --- | --- | --- | --- | --- | --- | --- | --- | --- |
| **A** | 1X PBS-2% BSA Only | | | | | | 50 pM | | | 1X PBS-2% BSA Only | | | | | | | | |
| **B** | High-C | S1 | S3 | S5 | S7 | | 100 pM | | | S9 | | S11 | | S13 | | S15 | | Low-C |
| **C** |  |  |  |  |  |  | 200 pM | | |  |  |  |  |  |  |  |  |  |
| **D** | Mid-C |  |  |  |  |  | 400 pM | | |  |  |  |  |  |  |  |  | Mid-C |
| **E** |  | S2 | S4 | S6 | S8 | | 800 pM | | | S10 | | S12 | | S14 | | S16 | |  |
| **F** | Low-C |  |  |  |  |  | 1600 pM | | |  |  |  |  |  |  |  |  | High-C |
| **G** |  |  |  |  |  |  | 3200 pM | | |  |  |  |  |  |  |  |  |  |
| **H** | 1X PBS-2% BSA Only | | | | | | 6400 pM | | | 1X PBS-2% BSA Only | | | | | | | | |
|  |  |  | | | |  | | |  | | | | | | | | |  |
|  | Control Samples | Unknown Samples, Triplicate | | | | Total H2AX Peptide Standards,  Duplicate | | | Unknown Samples, Triplicate | | | | | | | | | Control Samples |

S1 through S16 are unknown sample (S) wells in triplicate.

1. Sample Lysate Preparation

Unknown samples should be processed following SOP340520-Biopsy Specimen Processing for the DCTD Immunoassay (<http://dctd.cancer.gov/ResearchResources/ResearchResources-biomarkers.htm>) with the following **modifications**:

- 1. SOP340520 sample lysis references use of an 18-g needle biopsy. For preclinical sample processing, a similar tissue piece would weigh approximately 10 mg.
  2. In SOP340520, add 1 tablet PhosSTOP (Roche Applied Science, Cat#: 04906837001) per 10 mL Cell Extraction Buffer (CEB).

These lysis procedures are compatible with the ELISA protocol outlined in the following operating procedures.

1. elisa pROTOCOL
   1. **Key Reagents**

It is recommended to record the lot numbers, stock reagent concentration, and expiration dates for the Key Reagents in the lab record.

- - 1. **Total H2AX Peptide Standard**: Prepare as a 33000 pM stock solution. Aliquot in sufficient volumes for one 96-well plate; store at -80°C for ≤ 1 y.
    2. **Tumor Lysate Control**: Store at -80°C for up to 1 y. Lysates prepared to target Low, Medium and High total H2AX ranges.
    3. **H2A mAb**: Stock solution qualified from the manufacturer. Dilutions needed for assay performance for specific lot numbers of antibody will need to be determined by the assay site and should be matched to the pAb. Aliquot in sufficient volumes for one 96-well plate; store at ‑20°C for ≤ 6 mo.
    4. **H2AX pAb**: Stock solution qualified from the manufacturer. Dilutions needed for assay performance for specific lot numbers of antibody will need to be determined by the assay site and should be matched to the mAb Aliquot in sufficient volumes for one 96-well plate; store at ‑20°C for ≤ 6 mo.
    5. **Goat Anti-Rabbit HRP-Conjugated pAb**: Prepare a 1 mg/mL stock solution in HRP Stabilizer. Aliquot in sufficient volumes for three 96-well plates; store at 2°C to 8°C for ≤ 1 y.
    6. **Chemiluminescent Substrate Solutions**: Stock solutions (Peroxide and Pico Luminol/Enhancer Solutions) qualified from the manufacturer. Store at room temperature (25°C ± 3°C) for ≤ 3 mo. Protect from light during storage and use.
    7. **Reacti-Bind White Opaque 96-well Plate**: Store at 25°C ± 3°C away from volatile chemicals.
  1. **Plate Map and Buffer Preparation**
     1. Based on the number of unknown samples to be analyzed, generate a Plate Map (example, Step 2.0) to define the location and replicates of unknown samples, tumor lysate controls, and total H2AX peptide standards. Samples from a single experiment should be analyzed on one 96-well plate, not split over two, to ensure consistent sample handling.
     2. Once the number of wells is known, determine the amount of reagents required for the assay.

**IMPORTANT: For all of the following wash and aspiration steps, do not let the wells dry out.**

- 1. **Plate Preparation**
     1. Prepare 11 mL **H2A mAb** **Coating Solution** at a final concentration of 4µg/mL in 0.1 M pH9.6 carbonate buffer for the assay. This is sufficient for one 96-well plate (preparing enough for 110 wells). Thaw antibody immediately prior to dilution; do not allow sitting for extended periods upon thawing.
        1. If more than one 96-well plate is to be coated, pool the aliquots and then dilute appropriately. This will ensure that all plates are exposed to identical coating antibody. Discard excess diluted antibody.
     2. Add 100 µL of the **H2A mAb** **Coating Solution** per well using a multichannel pipettor, cover the plate with an acetate sheet, and incubate at 37°C for 2 h.
        1. Alternatively, the plate can be incubated overnight at 2°C to 8°C.
     3. Following incubation with the **H2A mAb** **Coating Solution**, aspirate the plate using a plate washer (for the BioTek Plate Washer, use the *Aspirate* program). After aspiration, tap the plate on paper towels to remove any residual liquid.
     4. Add 250 µL of SuperBlock to each well. Cover the plate with an acetate sheet and incubate at 37°C for 1 to 1.5 h.
        1. After blocking, move plate to 25°C ± 3°C until washing step (Step 4.7.1).
        2. Once coated plates have been blocked they can be stored at 2°C to 8°C for up to one week; do not let wells dry out.
  2. **Prepare Working Dilutions of Unknown Tumor Lysates**
     1. Stock lysates and protein concentration for tumors are prepared according to SOP340520.
     2. Tumor stock lysates with total protein concentration of < **0.2 μg/µL** should not be used in the total H2AX Immunoassay.
     3. Based on the protein measurements for the **stock tumor lysate** (μg/μL), prepare the **Working Lysates** in total H2AX assay diluent on ice for use in the total H2AX Immunoassay. Do not pipette less than 5 µL. If the calculations below yield volumes of stock lysate less than 5 µL, prepare sufficient volume of a 1:2 to 1:5 pre-dilution of the lysate before proceeding.
        1. For unknown stock lysates with stock protein concentrations > **0.75 µg/µL:**
- Prepare 10 µL of a 0.75 µg/µL **Working Lysate** as follows:

| 0.75 µg/µL  **Working Lysate** | * | 10 µL | = | XX μL Vol. Stock Lysate to use |
| --- | --- | --- | --- | --- |
| XXX µg/µL  Conc. Stock Lysate | | |  |  |
|  |  |  |  | |

- In a labeled 1.5 mL tube, add sufficient total H2AX assay diluent to the calculated volume of stock lysate needed to bring total volume to 10 µL.
- Add 65 µL of 1X PBS-2% BSA to generate 75 µL of 0.1 µg/µL **Diluted Working Lysate**.
  - - 1. For unknown stock lysates with stock protein concentrations **between 0.2 and 0.75 µg/µL**, no normalization is needed and directly dilute the stock lysate with 1X PBS-2% BSA for the **Diluted Working Lysate.**
  1. **Preparation of Unknown Tumor or PBMC Lysate Samples**
     1. Place all unknown samples to be assayed on ice. Each unknown tumor lysate will take up 3 sample spots (e.g., S1, S2, and S3).
     2. Tumor lysate samples
        1. For unknown **Working Lysates** with protein concentrations normalized to **0.75 µg/µL** and diluted at **0.1 µg/µL**,
        - Perform the following calculation to prepare 3 different lysate dilutions (0.5, 0.375, or 0.25 µg/well) in 100 μL total volume. For each **Diluted Working Lysate**, 1X PBS-2% BSA should be used to bring the total volume to 100 μL. This is sufficient volume to run each dilution in triplicate (plus 1 extra well). Clearly label all tubes with the sample number (e.g., S1, S2).

| (0.5, 0.375, or 0.25) µg/well  **Diluted Lysate** | * | 4 wells | = | (20, 15 or 10) μL **Working Lysate** |
| --- | --- | --- | --- | --- |
| 0.1 µg/µL  **Working Lysate** |  |  |  |  |
|  |  |  |  | |

- - - 1. For unknown **Working Lysates** with protein concentrations **between 0.2 and 0.75 µg/µL:**
      - Prepare 75 µL of a 0.1 µg/µL **Working Lysate** as follows:

| 0.1 µg/µL  **Working Lysate** | * | 75 µL | = | XX μL Vol. Stock Lysate to use |
| --- | --- | --- | --- | --- |
| XX µg/µL  Conc. Stock Lysate | | |  |  |
|  |  |  |  | |

- - - - In labeled 1.5-mL tube, add sufficient 1X PBS-2% BSA to the calculated volume of stock lysate needed to bring the total volume to 75 µL.
      - Perform the following calculation to prepare 3 different lysate dilutions (0.5, 0.375, or 0.25 µg/well) in 100 μL total volume. For each **Diluted Working Lysate**, 1X PBS-2% BSA should be used to bring the total volume to 100 μL. This is sufficient volume to run each dilution in triplicate (plus 1 extra well). Clearly label all tubes with the sample number (e.g., S1, S2).

| (0.5, 0.375, or 0.25) µg/well **Diluted Lysate** | | * | 4 wells | = | (20, 15 or 10) μL **Working Lysate** |
| --- | --- | --- | --- | --- | --- |
| 0.1 µg/µL  **Working Lysate** | |  |  |  |  |
|  |  | |  |  | |

- - - 1. Keep the **Diluted Lysate** on ice until use. Discard remaining **Working Lysate**.
    1. PBMC lysate samples
       1. Stock lysates for PBMCs (1 x 10^7^ cells/mL) are prepared according to SOP340506.
       2. Prepare PBMC working lysates by adding 7.5 μL of the stock lysate into 117.5 μL of 1X PBS-2% BSA (final 6 x 10^5^ cells/mL in 125 μL). Clearly label all tubes with the sample number (e.g., S7, S8).
       3. Lysates will be diluted an additional 3‑fold with 1X PBS-2% BSA once loaded into the 96-well plate yielding a relative load of 1.5 x 10^4^ cells/well.
       4. Keep all the samples on ice until use.
  1. **Preparation of H2AX Standards and Tumor Lysate Controls**
     1. Preparation of total H2AX standards; run in duplicate
        1. For one 96-well plate, retrieve one H2AX recombinant standard working stock solution tube (330000 pM) from the -80°C freezer and thaw on ice. Vortex and mix by inverting 5-8 times before use. Label eight 1.5-mL Sarstedt tubes, numbered 1 through 8, for the H2AX recombinant standards. Prepare a 9^th^ tube of just 1X PBS-2% BSA to load into the background wells.
        2. Prepare the H2AX recombinant standards by serial dilution with concentrations ranging from 19200 to 150 pM in 1X PBS-2% BSA.
        3. Standards will be diluted an additional 3‑fold when added to the 96-well plate to generate a reference curve ranging from 6400 to 50 pM total H2AX recombinant standard.
        4. Keep standards on ice until use. Only make enough standards for the assay and discard any excess.
     2. Preparation of tumor lysate controls; run twice on plate in duplicate
        1. For one 96-well plate, retrieve one of each High-C, Mid-C and Low-C tumor lysate control vial from the -80°C freezer and thaw on ice. Controls are provided at a concentration ready for use in the assay and no further dilution is required. Vortex and mix by inverting 5-8 times before use.
        2. Keep controls on ice until use. Controls will be diluted 3‑fold with 1X PBS-2% BSA once loaded into the 96-well plate.
  2. **H2AX Protein Capture**
     1. Following incubation with SuperBlock, the plates are aspirated and washed once with 350 µL of 1X PBS-0.1% Tween using a plate washer.

For the BioTek Microplate Washer, the settings are:

| **METHOD** |  |
| --- | --- |
| Number of Cycles: | **1** |
| Soak/Shake: | **No** |
| **DISPENSE** |  |
| Dispense Volume: | **350 µL/well** |
| Dispense Flow Rate: | **06** |
| Dispense Height: | **120 (15.240 mm)** |
| Horizontal DISP POS: | **00 (0.000 mm)** |
| Bottom Wash First: | **No** |
| Prime Before Start: | **No** |
| **ASPIRATE** |  |
| Aspirate Height: | **031 (3.937 mm)** |
| Horizontal ASPR POS: | **-20 (-0.914 mm)** |
| Aspiration Rate: | **05 (6.4 mm/sec)** |
| Aspirate Delay: | **1000 MSec** |
| Crosswise ASPIR: | **No** |
| Final Aspiration: | **Yes** |
| Final Aspirate Delay: | **1000 MSec** |
|  |  |

- - 1. After the wash, tap the plate on paper towels to remove residual buffer. Proceed immediately to the next step; do not allow the plate to dry out.
    2. Immediately, add 50 µL of 1X PBS-2% BSA containing 100 ng/well of Protamine to each well using a multichannel pipettor. Each well will hold a final volume of 75 μL after sample addition.
    3. Use the Plate Map as a guide to set up the 96-well plate for incubation with clinical samples, total H2AX standards, and assay controls. Pipette reagents in the following order; **do not deviate** from order of addition:

| **Order** | **Sample/Reagent and Volume** |
| --- | --- |
| 1 | 25 μL of specified concentrations of H2AX standards into designated duplicate wells. Load the lowest concentration first. |
| 2 | 25 μL of each unknown sample into designated triplicate wells. |
| 3 | 25 μL each of assay control (Low-C, Mid-C, and High-C) into both sets of designated duplicate wells. |
| 4 | 25 μL of additional 1X PBS-2% BSA into each of the Background wells. |

- - 1. Cover the plate with an acetate sheet and incubate at 2°C to 8°C for 18 ± 2 h.
  1. **H2AX Detection (next day)**
     1. Prepare a sufficient amount of the H2AX rabbit pAb 15 min before washing the plate (next step) that has been incubating with samples.
        1. Prepare 11 mL H2AX rabbit pAb working solution at a final concentration of 2µg/mL in 1X PBS-2% BSA supplemented with 1µL/mL mouse serum. This is sufficient for one 96-well plate (preparing enough for 110 wells). Thaw antibody immediately prior to dilution; do not allow sitting for extended periods upon thawing.
        2. Allow the prepared H2AX rabbit pAb to incubate for 15 min at 25°C ± 3°C.
     2. After the 18-h incubation is complete, aspirate and wash the wells 4 times with 350 µL of 1X PBS-0.1% Tween (same wash program as Step 4.7.1, except run for 4 cycles).
     3. After the wash, tap the plate on paper towels to remove residual Wash Buffer. Proceed immediately to the next step; do not allow the plate to dry out.
     4. Add 100 µL of the H2AX rabbit pAb working solution per well using a multichannel pipettor, cover the plate with an acetate sheet, and incubate for 2 to 2.5 h at 25°C ± 3°C. Discard residual working solution.
     5. 15 min before the incubation with the H2AX rabbit pAb is complete, prepare a sufficient amount of HRP conjugate for the assay.
        1. Prepare 11 mL HRP conjugate working solution at a final concentration of 1 µg/mL diluted in 1X PBS-2% BSA supplemented with 1µL/mL mouse serum. This is sufficient for one 96-well plate (preparing enough for 110 wells).
        2. Allow the prepared HRP conjugate to incubate in the dark at 25°C ± 3°C for 15 min.
     6. After the 2 to 2.5 h incubation with the H2AX rabbit pAb is complete, aspirate and wash the wells 4 times with 350 µL of 1X PBS-0.1% Tween (same wash program as Step 4.7.1, except run for 4 cycles). Tap plate on paper towels to remove residual liquid and proceed immediately to the next step.
     7. Add 100 µL of the HRP conjugate working solution per well using a multichannel pipettor. Cover the plate with an acetate sheet and incubate in the dark for 1 to 1.5 h at 25°C ± 3°C. Discard residual working solution.
  2. **Signal Detection**
     1. Turn on the Tecan Infinite Plate Reader at least 30 min before use. For luminescence optical density readings, the plate reader should be set to the following reading parameters:

| Shaking duration: | **5 sec** |
| --- | --- |
| Mode: | **linear** |
| Amplitude: | **1 mm** |
| Attenuation: | **OD1** |
| Integration Time: | **100 ms** |
|  |  |

- - 1. Just before the HRP conjugate incubation is finished, prepare 11 mL SuperSignal ELISA Pico Chemiluminescent Substrate Solution (5.5mL of Pico Stable Peroxide and 5.5mL of Pico Luminol/Enhancer). This must be made up immediately before use, kept in the dark, and at a sufficient volume for the assay.
    2. After the 1 to 1.5 h HRP conjugate incubation is complete, aspirate and wash the wells 4 times with 350 µL of 1X PBS-0.1% Tween (same wash program as SOP Step 8.7.1, except run for 4 cycles). Tap plate on a paper towel to remove excess buffer and proceed immediately to the next step.
    3. Add 100 µL of the freshly made Substrate Solution per well with a multichannel pipettor and avoid bright light.
    4. The first chemiluminescence reading should be within 2 min of substrate addition.
       1. If the signal is too high from the initial reading, wait 5 min and read the plate again at the same instrument setting. Continue reading until the RLU signal is on scale.
    5. Save the resulting readings as an Excel file.
    6. Use the RLU (relative light unit) values from the Excel file to generate the H2AX standard curve.
    7. Use the standard curve to convert RLU readings of the unknowns to H2AX readings in pM.

1. qUALITY cONTROL Recommendations
   1. **Background Well QC**
      1. Use the Plate Map for the identification of background wells that are to be used in QC determination. A total of 14 wells are used for background determination; the 4 corner wells and 2 adjacent to the high standard are not used for background level calculation.
      2. A ± 2 SD criterion is applied to the initial 14-well dataset to identify outliers.
         1. If a background well RLU value is ≥ 2 SD from the mean, delete that value from the background dataset cells.
      3. Once all wells that were ≥ 2 SD from the initial background dataset mean have been deleted, the %CV for the background wells must be < 20%.
         1. If the %CV for the background wells is < 20%, the assay passes QC.
         2. If the %CV is ≥ 20%, the **Assay Fails QC**, do not continue with the analysis. Rerun the assay with fresh reagents.
   2. **Standard Curve QC**

If at any point, the “**Assay Fails QC**,” do not continue with the analysis. State in the lab record the reason for assay failure. Rerun the assay with fresh reagents.

- - 1. **Low Standard QC and LLQ Assignment:**
    - In order to use the 50 to 100 pM range of the standard curve, the mean RLU readout of the 50 pM standard must be ≥ 3 SD above the mean RLU readout of the background; this value is referred to as the LLQ-RLU.
    - If the 50 pM standard fails, then the mean RLU readout of the 100 pM standard must be ≥ 3 SD above the mean RLU readout of the background.
    - If the 100 pM standard also fails, the **Assay Fails QC**.
    - The lowest passing standard is assigned as the LLQ (pM) for the assay.
    1. **Signal-to-background (S/B) ratio QC and ULQ Assignment:**
    - The ratio for the lowest passing standard (50 or 100 pM) RLU readout to the mean RLU readout of the background must be ≥ 1.1. If not, the **Assay Fails QC**.
    - The ratio of the highest standard RLU readout (6400 pM) to the mean RLU of the background must be ≥ 15. If not, the **Assay Fails QC**.
    - If the high standard passes QC, it is assigned as the ULQ (pM) for the assay.
  1. **Control Samples**
     1. The QC determination for the control samples should have the following criteria:
     - At least one control at each level (Low-, Mid-, and High-C) must have a CV of < 20% for the replicate wells.
     - At least one control at each level and at least 4 of 6 controls overall must fall within the defined total H2AX pM range provided for the **specific lot** of critical reagent.
     1. If any of these criteria are not met, the **Assay Fails QC**. Rerun the assay with fresh reagents.
  2. **Unknown Sample Replicate QC and LLQ/ULQ QC**
     1. Triplicate repeats for each sample must have a CV < 20%.
     2. Review the average total H2AX levels and identify any values that are < LLQ or > ULQ.
        1. If a sample is > ULQ and there is sufficient sample volume, it can be re-run with fresh reagents at a 2-fold lower protein load/well. If a sample is < LLQ and there is sufficient sample volume, it can be re-run at a 2-fold higher protein load/well.
